# Adherence to the GOLD Guidelines in Primary Care: Data from the Swiss COPD Cohort

**DOI:** 10.3390/jcm12206636

**Published:** 2023-10-20

**Authors:** Veronika Mangold, Maria Boesing, Camille Berset, Pierre-Olivier Bridevaux, Thomas Geiser, Ladina Joos Zellweger, Malcolm Kohler, Giorgia Lüthi-Corridori, Sabrina Maier, David Miedinger, Robert Thurnheer, Christophe von Garnier, Jörg Daniel Leuppi

**Affiliations:** 1 University Center of Internal Medicine, Cantonal Hospital Baselland, 4410 Liestal, Switzerland; 2 Faculty of Medicine, University of Basel, 4056 Basel, Switzerland; 3 Clinic of Pneumology, Hospital of Valais, University of Geneva, 1205 Geneva, Switzerland; 4 Department of Pulmonary Medicine and Allergology, University Hospital, University of Bern, 3010 Bern, Switzerland; 5 Department of Pneumology, Claraspital, 4058 Basel, Switzerland; 6 Department of Pneumology, University Hospital Zürich, 8091 Zürich, Switzerland; 7 Clinic of Medicine, Department of Pneumology, Cantonal Hospital Münsterlingen, 8596 Münsterlingen, Switzerland; 8 Division of Pulmonology, Department of Medicine, University Hospital Lausanne, CHUV, University of Lausanne, 1011 Lausanne, Switzerland

**Keywords:** COPD, GOLD guidelines, adherence, primary care, inhaled corticosteroids, bronchodilators

## Abstract

(1) Introduction: Chronic obstructive pulmonary disease (COPD) and its associated morbidity and mortality are a global burden on both affected patients and healthcare systems. The Global Initiative for Chronic Obstructive Lung Disease (GOLD) issues guidelines with the aim of improving COPD management. Previous studies reported significant variability in adherence to these recommendations. The objective of this study was to evaluate Swiss primary practitioners’ adherence to the GOLD guidelines for the pharmacological treatment of stable COPD. (2) Methods: We studied patients who were included in the Swiss COPD cohort study, an ongoing prospective study in a primary care setting, between 2015 and 2022. The key inclusion criteria are age ≥ 40 years, FEV1/FVC ratio < 70%, and a smoking history of at least 20 pack-years. Adherence to the GOLD guidelines was assessed per visit and over time. (3) Results: The data of 225 COPD patients (mean age 67 ± 9 years, 64% male) and their respective 1163 visits were analyzed. In 65% of visits (726/1121), treatment was prescribed according to the GOLD guidelines. Non-adherence was most common in GOLD groups A and B (64% and 33%) and mainly consisted of over-treatment (two long-acting bronchodilators in group A (98/195, 50%) and ICS in groups A (21/195, 11%) and B (198/808, 25%)). In group D, the prescriptions conformed with the guidelines in 99% of cases (109/108). Guideline adherence was associated with high symptom load (COPD Assessment Test) (OR 1.04, *p* = 0.002), high number of exacerbations (OR = 2.07, *p* < 0.001), asthma overlap (OR 3.36, *p* = 0.049), and diabetes mellitus (OR 2.82, *p* = 0.045). (4) Conclusion: These results confirm a conflict between the GOLD recommendations and primary practice, mainly concerning over-treatment in GOLD groups A and B. Patients with high symptom load, high exacerbation risk, asthma overlap, and diabetes mellitus are more likely to be treated in conformity with the guidelines. Further research is needed to uncover the reasons for the discrepancies and to design strategies for improvement.

## 1. Introduction

Chronic Obstructive Pulmonary Disease (COPD) is a frequent, progressive, and preventable disease. It is an inflammatory lung disease, mostly caused by tobacco use [1]. However, in many countries, outdoor, occupational, and indoor air pollution represent other significant risk factors for COPD [2]. The disease is characterized by an airflow obstruction that is not completely reversible, dyspnea, and a chronic cough [3]. 

**Figure 1:**
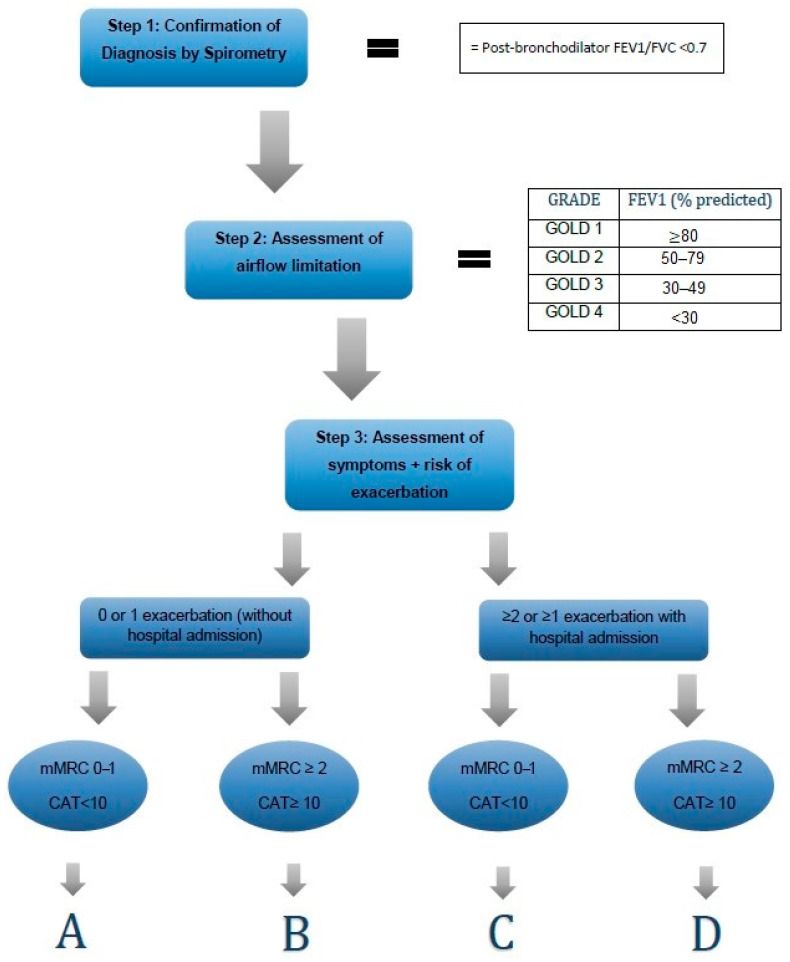
The ABCD assessment tool [9]. FEV1: Forced expiratory volume in one second; FVC: forced vital capacity; GOLD: Global Initiative for Chronic Obstructive Lung Disease; CAT: COPD Assessment Test; mMRC: modified Medical Research Council score.

More than 5% of the worldwide population suffers from COPD, with 90% of them being smokers or former smokers [4]. COPD is associated with increased morbidity and mortality [5,6]. As the fourth leading cause of death worldwide [6], COPD presents a major global burden and an important public health challenge. However, COPD symptoms can be treated successfully, and smoking cessation can slow down or even stop the progression of the disease [7]. COPD can be classified into grades GOLD 1–4 according to airflow limitation, measured by forced expiratory volume in one second (FEV1) (see Figure 1).

The Global Initiative for Chronic Obstructive Lung Disease (GOLD) aims to increase awareness of COPD in primary care, as well as improve its diagnosis and treatment [8,9]. Being a disease mostly managed in a general practice setting [10], the application of the GOLD guidelines among general practitioners is crucial. In Switzerland, a large proportion of COPD patients are managed by primary care pneumologists, who make treatment decisions related to COPD. Thus, primary care pneumologists’ adherence to the GOLD guidelines is equally important.

However, the GOLD guidelines have changed on a regular basis since the project began in 2006. They have been updated every 5–6 years (2006, 2011, 2017, 2023) and revised yearly. The fact that airflow limitation alone is not a sufficient parameter to reflect the real severity of the disease led to a major revision in 2011, including a new classification system for COPD patients and a recommendation to base assessment on both the symptoms and the risk of future exacerbations, as presented in Figure 1 [9]. The conclusion was to group patients into four groups: A (low risk, few symptoms), B (low risk, more symptoms), C (high risk, few symptoms), and D (high risk, more symptoms). Another major revision of the GOLD guidelines in 2017 redistributed COPD patients by eliminating the previous classification that was based on the severity of airflow obstruction [11].

Until the update of the GOLD therapy guidelines in 2017, the treatment recommendations were divided into first and second choices. Since 2017, there have been no first- and second-choice recommendations, but solely recommendations with different options of equal priority. Table 1 shows an overview of the recommended treatment of stable COPD using the 2011 and 2017 GOLD guidelines. 

The pharmacological treatment recommendations from the 2017 GOLD report can be summarized as follows: for group A, the recommended therapy includes therapy with any bronchodilator as a minimum; group B includes at least one long-acting bronchodilator; and groups C and D include therapy with a long-acting muscarinic antagonist (LAMA). In the case of insufficient symptoms or exacerbation control, this should be either combined with a long-acting beta-agonist (LABA) or switched to a combination of LABA with an inhaled corticosteroid (ICS) (LABA/ICS). In group D, the guidelines suggest the extension to an LABA/LAMA/ICS combination or a switch to an LABA/ICS combination for patients with further exacerbations. If needed, further therapy escalation can include the addition of a phosphodiesterase-4 inhibitor (roflumilast), a macrolide, or stopping the ICS. Due to the lack of benefits and a high rate of systemic complications, oral glucocorticoids are not recommended for the chronic daily treatment of stable COPD [11].

Even though the GOLD guidelines are well communicated, publicly available, and updated every year, their implementation in primary practice is not consistent. Previous studies indicated an insufficient adherence of general practitioners (GPs) to the GOLD guidelines [13,14,15,16,17]. COPD diagnosis and the monitoring of the development of the disease is often challenging, wherefore it demands confirmation using spirometry [9]. However, doctors in primary care mostly do not perform spirometry routinely, due to lack a of office spirometry devices or a lack of training [18]. According to a previous analysis of data from the Swiss COPD Cohort, 21.5% of the patients treated in primary care did not comply with the spirometry diagnostic criteria for COPD [19]. Furthermore, a Danish cross-sectional survey in 124 primary care practices showed that out of 1716 COPD patients, only 7% had their dyspnea assessed on the modified Medical Research Council Dyspnoea Scale (mMRC) [20]. Another study pointed out that among 57 general practitioners with more than 30 000 COPD patients in the Balearic Islands, Spain, the mMRC or the COPD Assessment Test (CAT) were recorded in less than 0.5% of the patients [21]. There is evidence that using collected information, such as spirometry data, disease progression data, and therapeutic measures, improves both the patients’ management and self-management [18]. Additionally, the assessment of the mMRC or the CAT is crucial for the recommended classification into groups A-D (see Figure 1), facilitating evidence-based treatment decisions. 

Recent research shows that most COPD patients in the primary care sector of the United Kingdom are treated with inhaled corticosteroids (ICS), independent of the GOLD classifications [22]. In addition, a study from Taiwan confirmed a discordance between pharmacological therapy and the GOLD guidelines among COPD patients in Taiwanese hospitals, especially after the guideline update in 2017 [12]. Previous data from the Swiss COPD cohort showed that in 2010, guideline non-conformity particularly concerned the lack of use of short-acting bronchodilators, pulmonary rehabilitation, and regular exercise [13]. To summarize, from a guideline perspective, COPD is often misdiagnosed or treated inappropriately [23], which may result in increased morbidity and mortality associated with COPD [24].

## 2. Materials and Methods

### 2.1. Study Design and Objectives

The Swiss COPD Cohort Study is an ongoing, questionnaire-based, prospective cohort study, developed to investigate the course of COPD in patients managed in primary care in Switzerland. After inclusion into the study, patients are followed up in six-monthly study visits at their GP or pneumologist. 

The aim of this sub-project was to evaluate if patients received COPD therapy based on the current GOLD guidelines during the study duration (GOLD guidelines reviewed in 2011 and 2017, respectively), as a follow-up on previously published data [13]. In addition, we aimed to identify potential patient- and disease-related factors that are associated with guideline conformity and investigate if guideline adherence changed over time.

### 2.2. Study Population

The Swiss COPD Cohort Study includes COPD patients from northern and western Switzerland, managed by GPs and pneumologists in the primary care sector. Patients with mild to very severe COPD (GOLD groups A–D) are enrolled in the cohort study. Recruitment started in 2007 and since then almost 100 GPs have been recruiting patients for the study. The inclusion criteria are as follows:

- Confirmed COPD diagnosis (FEV1/FVC < 70%, before and after inhalation of a bronchodilator);
- Age ≥ 40 years;
- Current smokers or former smokers of at least 20 pack-years;
- Informed consent.

This sub-project included patients who were enrolled in the Swiss Cohort Study between January 2015 and December 2022. There were no exclusion criteria.

### 2.3. Study Procedures

All study visits are performed in a decentralized manner by the patient’s managing GP or pneumologist—depending on which one is mainly managing the patient’s COPD. Upon enrolment into the study, patients undergo a baseline visit where demographic data, patient history including exacerbation history, current symptoms (by COPD assessment test (CAT) and modified medical research council score (mMRC)), lung function (by spirometry), and current COPD medication are assessed. Patients’ follow-up visits occur at six-month intervals. During each follow-up visit, recent exacerbation history, symptoms (cough, sputum, CAT and mMRC), and lung function are assessed. With the Swiss COPD cohort being an observational study, all treatment decisions during the study period are left at the discretion of the COPD managing primary care physician.

### 2.4. Outcomes

This study focused on assessing adherence to GOLD guidelines in the pharmacological treatment prescribed by GPs. Primarily, at every visit, we assessed adherence of the included patients within the study period, based on the recommendations given in Table 1 for SABA, SAMA, LABA, LAMA, and ICS. Additionally, prescribed short-acting bronchodilators were always considered adherent, assuming they were prescribed as rescue medication for immediate symptom relief. Given that guideline adherence was assessed for every single visit, variations in adherence were possible across one single patient’s multiple visits. In order to summarize the outcome at a patient level, we defined a patient as “treated according to guidelines”, if the managing physician had prescribed guideline-conforming treatment in at least 70% of the patient’s study visits. The classification into groups A-D was based on the GOLD definition using the results of mMRC and CAT, as well as the recent exacerbation history (see Figure 1). The classification was reassessed for every follow-up visit.

### 2.5. Statistical Methods and Analysis

**Figure 2:**
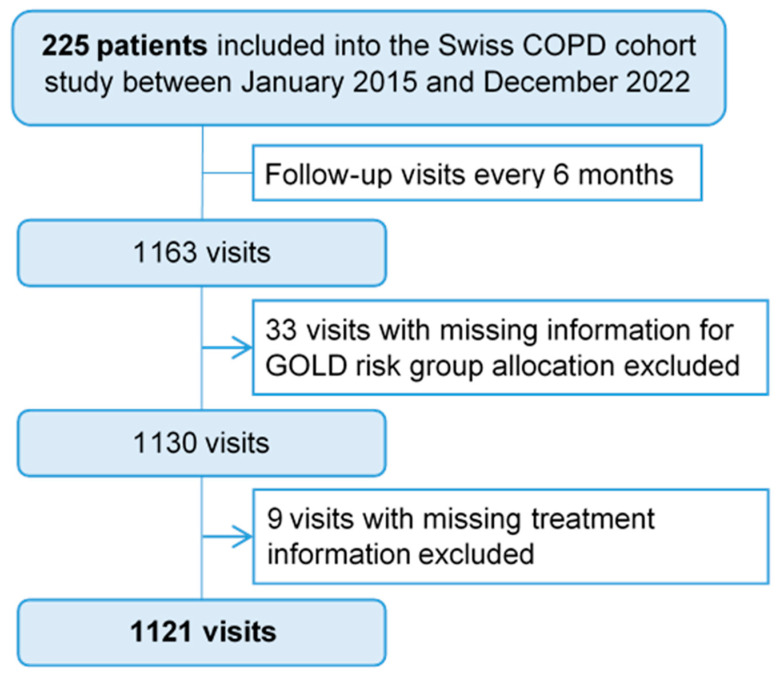
Flow chart for patient and visit selection process. COPD: Chronic Obstructive Pulmonary Disease; GOLD: Global Initiative for Chronic Obstructive Lung Disease.

Results are presented descriptively as absolute and relative frequencies for categorical variables, mean ± standard deviation (SD) for normally distributed variables, and median/interquartile range (IQR) for skewed variables. Multivariable logistic regression models were fitted to identify patient- and disease-related factors associated with guideline non-conformity. Missing data was indicated in all tables, where relevant. Specifically, 33 follow-up visits could not be assigned to a GOLD risk group due to missing mMRC and CAT data or exacerbation history. For an additional nine visits, guideline adherence could not be evaluated due to missing treatment data. These 42 visits were excluded from the presentation of treatment and adherence in figures and tables concerning treatment and guideline-adherence (see Figure 2).

## 3. Results

A flow chart with the patient and visit selection processes is presented in Figure 2. Altogether, 225 patients were enrolled in the study between 2015 and 2022, who attended 1163 visits. Demographic data and patient baseline characteristics are summarized in Table 2. The mean age was 66.8 years (±9.44, range 40–88) and 64% (n = 144) of the study population were men. More than two thirds of the patients (69%, n = 155) were classified into group GOLD B at baseline, followed by group A (17%, n = 39) and D (11%, n = 25). The most common comorbidity across all the groups was arterial hypertension (52%, n = 117).

**Figure 3:**
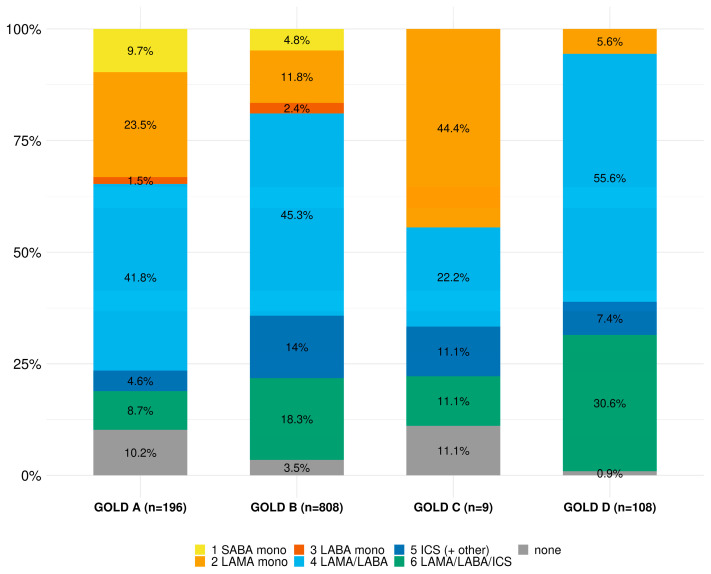
Pharmacological COPD treatment regimen in each GOLD group per visit (n = 1121 visits, 33 visits omitted due to missing GOLD group information, 9 visits omitted due to missing treatment information). GOLD: Global Initiative for Chronic Obstructive Lung Disease; SABA: short-acting β2-agonist; LABA: long-acting β2-agonist; LAMA: long-acting muscarinic antagonist; ICS: inhaled corticosteroid.

Due to missing documentation (33 pieces of missing GOLD group information, nine visits with missing treatment information), guideline adherence could not be evaluated in 42 out of 1163 visits (3.6%). Of the remaining 1121 visits, the prescribed pharmacological COPD treatment per visit in each GOLD group is presented in Figure 3 and any alternative/non-pharmacological therapy in Table 3. Within GOLD group A, a short-acting bronchodilator was only prescribed in 15% of the visits, while long-acting bronchodilators were prescribed more frequently (LABA 4%, LAMA 34%, LABA/ICS 12%, and LABA/LAMA 41%). In GOLD group B, in about one-third of visits, inhaled corticosteroids were prescribed (ICS 6% and LABA/ICS 26%). The LABA/LAMA combination was prescribed in almost half of the visits (49%). In GOLD group C, a long-acting bronchodilator was prescribed in all visits (LAMA 56%, LABA/ICS 22%, and LABA/LAMA 22%). Visits within GOLD group D had the highest percentage of dual bronchodilator prescriptions LABA/LAMA (68%), systemic steroids (5%), and oxygen therapy (21%). The influenza vaccination status was positive in 77% of the visits overall.

**Figure 4:**
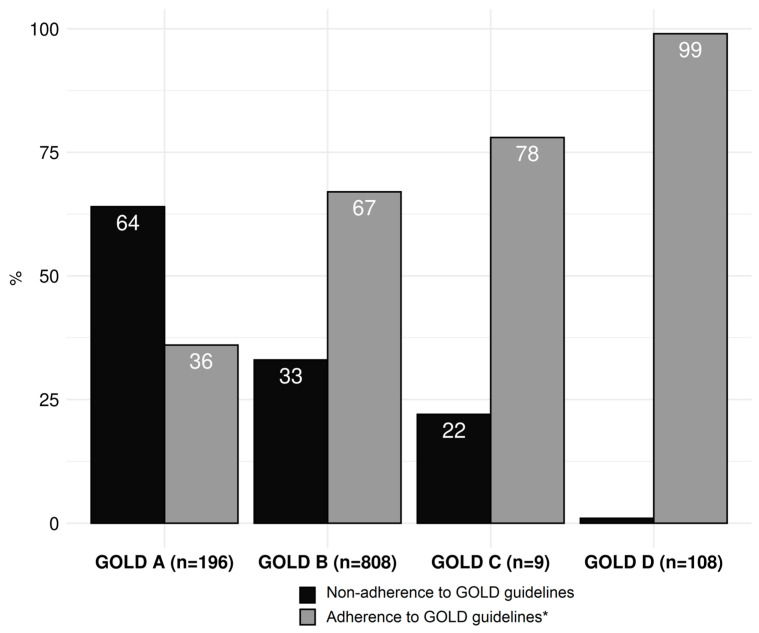
Adherence to GOLD guidelines for each GOLD group per visit in percent (n = 1121 visits, 33 visits omitted due to missing GOLD group information, 9 visits omitted due to missing treatment information). * ICS was considered adherent in groups A and B if the patient had a documented asthma diagnosis.

Figure 4 presents conformity and non-conformity rates with the GOLD guidelines for the treatment of stable COPD for each GOLD group per visit. In patients with a documented asthma comorbidity, ICS were always considered adherent to the guidelines, regardless of the GOLD risk group. However, if a patient in GOLD group A or B had no documented asthma diagnosis, ICS treatment was considered to be non-adherent to the guidelines. The prescriptions made by the treating doctors did not correspond to the guidelines in 395 visits (35%). GOLD group A had the highest rate of non-conforming prescriptions (n = 125, 64%), followed by GOLD group B (n = 267, 33%), while prescriptions in GOLD group D were mostly accomplished according to the guidelines (n = 108, 99%). The main driver for non-conformity in GOLD group A was the prescription of dual therapy with LAMA/LABA (n = 98, 50%, Table 4). In GOLD group B, ICS were prescribed in 198 visits (25%) against the GOLD recommendations and when no overlapping asthma diagnosis was documented (Table 4).

**Figure 5:**
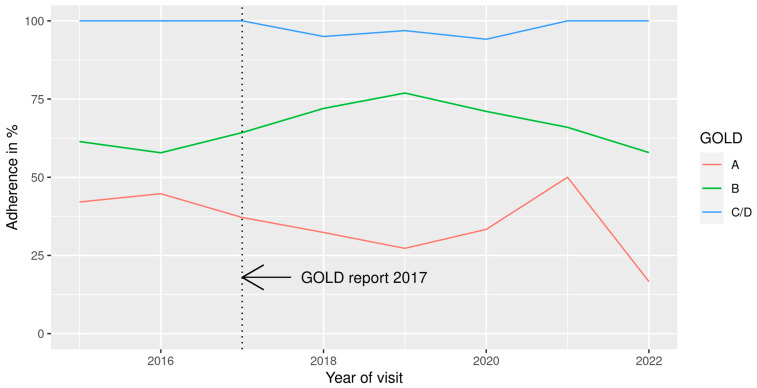
Guideline adherence in each GOLD group per visit over time (2015–2022) (n = 1121 visits, 33 visits omitted due to missing GOLD group information, 9 visits omitted due to missing treatment information). Due to low number of visits, GOLD groups C and D were combined into C/D.

Figure 5 presents the adherence to the guidelines for each GOLD risk group over time. In GOLD risk groups C and D, guideline adherence was better than in GOLD groups A and B throughout, and only marginal changes were observed from the years 2015 to 2022. In GOLD group B, guideline adherence improved between 2016 and 2019 (from 58% to 77%) and dropped again continuously until 2022 (to 58%). In GOLD risk group A, guideline adherence was best in 2021 (50%), whereas a substantial drop to 17% was observed in 2022.

**Figure 6:**
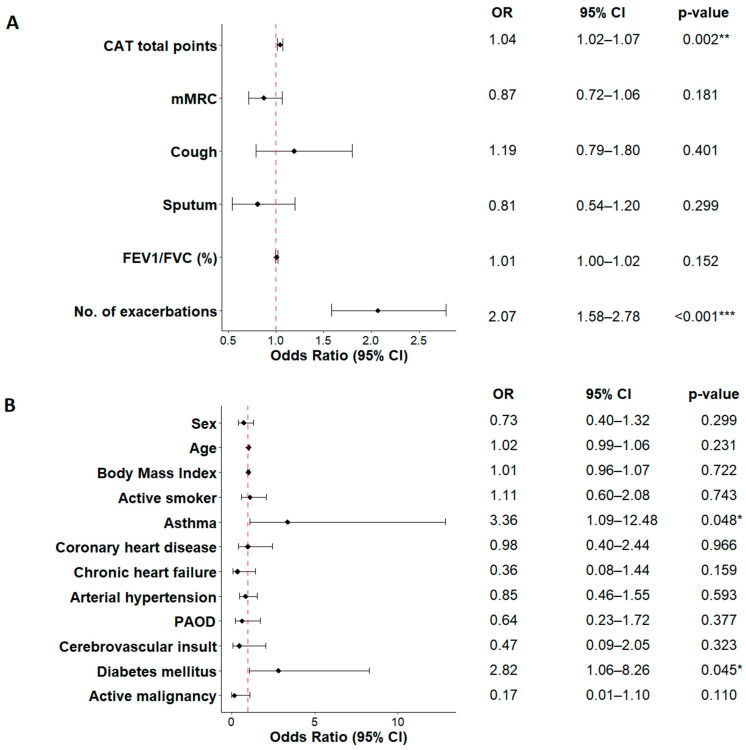
Predictors for guideline adherence (multivariable logistic regression). Red dashed line represents odds ratio of 1. (**A**): On visit basis (n = 1121 visits, 33 visits omitted due to missing GOLD group information, 9 visits omitted due to missing treatment information). (**B**): On patient basis (n = 225 patients). OR: Odds ratio; CI: Confidence interval; CAT: COPD Assessment Test; mMRC: modified Medical Research Council score; FEV1: forced expiratory volume in 1 s; FVC: forced vital capacity; PAOD: peripheral arterial occlusive disease. Significance codes: * *p* < 0.05, ** *p* < 0.01, *** *p* < 0.001.

The multivariable logistic regression revealed the following results (Figure 6). On a visit basis, guideline adherence was associated with a high symptom burden (CAT) (OR 1.04, *p* = 0.002), and a high number of exacerbations in the past 12 months (OR 2.07, *p* < 0.001) (Figure 6A). On a patient basis, guideline adherence was associated with asthma overlap (OR 3.36, *p* = 0.049) and diabetes mellitus (OR 2.82, *p* = 0.045) (Figure 6B).

## 4. Discussion

Our analysis of adherence to the GOLD guidelines in the treatment of COPD patients in primary care has three main findings. First, primary practitioners’ adherence to the GOLD guidelines for the pharmacological treatment of stable COPD is still poor, with guideline-conforming therapy only happening in 64% of all visits. Second, non-conformity to guidelines mainly consists of overtreatment in GOLD groups A and B, while group D patients are treated in line with the guidelines in 99% of the visits. Third, high symptom burden, high number of exacerbations, asthma overlap, and diabetes mellitus are associated with guideline adherence.

The refinement of the COPD classification into groups A–D in 2011 led to treatment recommendations that are based on symptoms and exacerbation risk [25]. When compared with previous findings, guideline adherence has improved since then. In 2010, Jochmann et al. reported an overall guideline non-adherence rate of 44%, when considering the prescription of ICS for co-morbid asthma acceptable [13]. Our data showed a lower non-adherence rate of 35%, which could indicate that the ABCD classification and the associated recommendations were more practicable and accepted among practitioners.

However, the discordance between the guidelines and primary care practice in GOLD groups A and B is still unacceptably high. Jochmann et al. found the highest rates of non-conforming prescriptions in GOLD stages I and II (45% and 54%, respectively) [13], while we found them in GOLD groups A and B (64% and 33% of visits, respectively). The results of a questionnaire-based study in Switzerland in 2017 reported similar numbers of non-conformity mainly consisting of overtreatment, the highest rates being in GOLD groups A and B [23]. In our cohort, the main discordances with the GOLD recommendations were also predominantly overtreatments with two long-acting bronchodilators (GOLD group A) and ICS (GOLD A and B). GOLD group A patients are COPD patients with a low symptom burden and a low risk for exacerbations—a single long-acting bronchodilator should provide sufficient symptom and exacerbation control in this patient group. However, some patients in GOLD group A may have had more symptoms and/or exacerbations in the past, which would justify the dual therapy approach. In this case, patients and practitioners may be rightly reluctant to a reduction in therapy. 

Overtreatment with ICS was reported by several previous studies [22,23,26]. While ICS is well-established and recommended in patients with asthma [27], the 2011 and 2017 GOLD guidelines recommend not prescribing it to patients in groups A and B, without an asthma diagnosis [9,11]. ICS treatment is associated with an increased pneumonia risk, along with other undesirable side effects [11,28]. Therefore, when initiating an ICS treatment in these groups, the benefits should be carefully weighed against the risks, especially in patients with a history of pneumonia and/or mycobacterial infection and low blood eosinophils [29]. 

Remarkably, in 20 out of 195 visits in GOLD group A (10%), practitioners prescribed no bronchodilator at all. In 71 out of 808 visits in group B (9%), no long-acting bronchodilator was prescribed. This undertreatment in moderate to severe COPD was previously described in a Canadian CAGE study [30]. Given the available evidence on the positive effect of bronchodilators on health status, symptom control, and exacerbation rate, the reasons and possible interventions to improve guideline adherence should be investigated further [31,32,33].

In GOLD group D, guideline adherence was exceptionally good in our cohort when compared with previous studies. While Marmy et al. reported 15.2% of undertreated patients in this group [23], our results show very strong guideline adherence of 99%. In addition, guideline conformity was associated with a high symptom burden and a high number of exacerbations in the past 12 months. These results may indicate that guidelines for this patient group are widely known, accepted, and confidently put into practice. However, since the recommendations for this group include almost all possible combinations of bronchodilators and ICS, clear overtreatment is hardly possible [9,11]. In this group, the practitioner’s challenge is to find each individual patient’s ideal substance combination, while only escalating the therapy further when necessary.

As shown in Figure 5, only marginal changes in good guideline adherence were observed in groups C and D over time. Furthermore, the release of the 2017 GOLD report did not seem to have any effect on guideline adherence in groups A and B. Guideline adherence in GOLD group B improved until 2019, only to drop again until 2022. GOLD group A showed the biggest variation in guideline adherence with a rise to 50% in 2021 and substantial drop to 17% in 2022. It is important to note that due to the COVID-19 pandemic, the number of performed visits dropped dramatically after 2019. While the overall number of performed visits ranged between 190 and 220 in the beginning of the observation period, only 70 visits were performed on average in each year of the pandemic. With patients that have the mildest form of COPD, this drop was particularly pronounced in GOLD group A, where only six visits were performed in the years 2021 and 2022. Therefore, we believe that the above-described variation in adherence in Figure 5 is mainly a result of the fluctuation of the underlying number of visits. However, the overall picture confirms the previously described tendency for overtreatment in GOLD groups A and B. On a visit level, we found an association of guideline adherence with high symptom burden (CAT) and high risk for exacerbation (Figure 6)—the criteria for the classification into GOLD group D. This association not only underlines the high guideline adherence in this group but also indicates that patients with either high symptom load or high risk for exacerbations (namely GOLD groups B and C) are mostly treated in line with the guidelines. When an asthma diagnosis was documented, we considered an ICS to be the acceptable treatment in all groups, which made overtreatment with regard to the guidelines more or less impossible in GOLD groups B, C, and D. Other studies investigating the topic excluded patients with an overlapping asthma diagnosis, in order not to overestimate the ICS overtreatment [34]. Patients with diabetes mellitus were also more likely to be treated in line with the guidelines. In our studied cohort, 27 patients (12%) were affected by diabetes mellitus, with similar proportions across GOLD groups A, B, and D. While previous studies reported a higher prevalence of diabetes mellitus in COPD patients (16% to 18%), the association with guideline adherence has not been described before [35,36]. One possible explanation could be that patients with diabetes visit their GP more frequently than others; therefore, they are more closely monitored, and treatment is adjusted more often. However, this hypothesis would have to be investigated further. 

Meanwhile, the 2023 GOLD report was published [29], with one of the main changes being another revision of the ABCD Assessment Tool to the ABE Assessment Tool, including the respective treatment recommendations (see Table A1, Appendix A) [29]. The reason for this was to acknowledge the high clinical relevance of exacerbations, independent of the level of symptoms. As seen in our cohort, COPD patients with low symptom burden and high risk for exacerbations (GOLD group C) are rare. The aggregation of groups C and D into group E may thus make recommendations even more practicable and accepted.

The objective of this study was to evaluate pharmacological COPD management and adherence to the GOLD guidelines among primary practitioners in Switzerland. Our study confirms the previously found discrepancies between prescription patterns and the internationally recognized GOLD guidelines. Besides the lack of GP familiarity with the current guidelines, other possible reasons are time constraints in daily practice (e.g., for the repeated re-assessment of the GOLD group), and a certain amount of skepticism among practitioners towards the efficacy of the recommended treatment, especially when overtreatment is practiced. Additionally, practitioner guideline conformity depends highly on patients’ individual adherence to the given prescriptions. Recent studies have also shown that involving the patient in setting up an individually customized therapy seems to improve patients’ adherence, which might also lead to guideline non-conforming prescriptions [37,38].

### Limitations

While the results of this study represent real-life insights into primary care practice, the study has some limitations. Practitioners who agree to take part in the Swiss COPD cohort study presumably have a certain interest in research and evidence-based treatment, which may have created a bias towards higher guideline adherence. In addition, all study-related procedures, including CAT, mMRC, and exacerbation history are performed by the respective treating practitioner in a decentralized manner. This may have resulted in subjective assessment, especially with regard to the ABCD classification. In this study, we only evaluated the adherence to pharmacologic treatment recommendations of stable COPD, while non-pharmacologic therapy options, such as physical activity and pulmonary rehabilitation were not taken into account. Furthermore, the individual reasons for guideline non-adherence were not investigated.

## 5. Conclusions

Although the GOLD guidelines are regularly revised and adapted in line with the latest evidence, adherence in primary care has only slightly improved in recent years. Overprescription of ICS in patients with low exacerbation risk (GOLD groups A and B) remains the most common therapeutic mistake, followed by the overtreatment of dual bronchodilator therapy in GOLD group A. The most frequent undertreatment was the non-prescription of a long-acting bronchodilator in GOLD group B. However, our results showed that patients with a high burden of disease are mostly treated according to the GOLD guidelines. The results of this study add to the evidence that COPD guidelines are not sufficiently put into practice. Further research is needed to obtain insight into the reasons for guideline non-adherence and to design effective strategies for its improvement.

## Figures and Tables

**Table 1:** Recommended treatment for stable COPD according to the 2011 and 2017 GOLD guidelines (modified from [12]).

Guidelines	GOLD 2011		GOLD 2017
GOLD Group	First Choice	Second Choice	Recommended
A	SABA or SAMA, or SABA/SAMA	LABA or LAMA	SABA or SAMA, or SABA/SAMA or LABA, or LAMA
B	LABA or LAMA	LABA/LAMA	LABA or LAMA or LABA/LAMA
C	ICS/LABA or LAMA	LABA/LAMA	LAMA or LABA/LAMA or ICS/LABA
D	ICS/LABA or LAMA	ICS/LABA/LAMA, LABA/LAMA, or LAMA/ICS	LAMA or LABA/LAMA or ICS/LABA, or ICS/LABA/LAMA, or LAMA/ICS

SABA: short acting beta-agonist; SAMA: short-acting muscarinic-antagonist; LABA: long-acting beta-agonist; LAMA: long-acting muscarinic antagonist; ICS: inhaled corticosteroid.

**Table 2:** Baseline characteristics (n = 225 patients).

Subjects, n (%)	Overall	GOLD A	GOLD B	GOLD C	GOLD D	Missing (%)
	225 (100)	39 (17.33)	155 (68.89)	6 (2.67)	25 (11.11)	
Age (years), mean ± SD	66.84 ± 9.44	66.41 ± 10.00	66.54 ± 9.23	70.17 ± 9.75	68.56 ± 10.01	0.0
Gender, n (%)						0.0
Male	144 (64.0)	32 (82.1)	91 (58.7)	6 (100.0)	15 (60.0)	
Female	81 (36.0)	7 (17.9)	64 (41.3)	0 (0.0)	10 (40.0)	
BMI, mean ± SD	26.64 ± 5.79	28.46 ± 6.06	26.32 ± 5.77	25.48 ± 3.88	26.09 ± 5.52	0.4
Smoking status						0.0
Current smokers, n (%)	129 (57.3)	21 (53.8)	92 (59.4)	3 (50.0)	13 (52.0)	
Ex-smokers, n (%)	96 (42.7)	18 (46.2)	63 (40.6)	3 (50.0)	12 (48.0)	
Pack-years, mean ± SD	47.98 ± 20.03	44.85 ± 15.57	47.36 ± 20.30	64.83 ± 17.78	52.64 ± 23.15	
Spirometry						
FEV_1_/FVC in %, mean ± SD	54.94 ± 10.01	59.47 ± 8.46	54.70 ± 9.95	51.14 ± 6.93	50.33 ± 10.89	0.0
Respiratory symptoms, n (%)						
Sputum	150 (67.0)	13 (33.3)	114 (74.0)	5 (83.3)	18 (72.0)	0.4
Cough	174 (78.0)	23 (59.0)	129 (83.8)	4 (80.0)	18 (72.0)	0.9
CAT, mean ± SD	15.82 ± 7.86	6.44 ± 2.16	18.08 ± 6.82	5.20 ± 2.28	18.76 ± 7.55	1.3
Dyspnoea mMRC 0	16 (7.2)	12 (30.8)	3 (1.9)	1 (16.7)	0 (0.0)	1.3
Dyspnoea mMRC 1	88 (39.6)	27 (69.2)	51 (32.9)	5 (83.3)	5 (22.7)	
Dyspnoea mMRC 2	81 (36.5)	0 (0.0)	71 (45.8)	0 (0.0)	10 (45.5)	
Dyspnoea mMRC 3	32 (14.4)	0 (0.0)	26 (16.8)	0 (0.0)	6 (27.3)	
Dyspnoea mMRC 4	5 (2.3)	0 (0.0)	4 (2.6)	0 (0.0)	1 (4.5)	
Comorbidities, n (%)						0.0
Asthma, n (%)	18 (8.0)	2 (5.1)	15 (9.7)	0 (0.0)	1 (4.0)	
CHD, n (%)	31 (13.8)	3 (7.7)	26 (16.8)	1 (16.7)	1 (4.0)	
Chronic heart failure, n (%)	10 (4.4)	1 (2.6)	9 (5.8)	0 (0.0)	0 (0.0)	
Hypertension, n (%)	117 (52.0)	19 (48.7)	82 (52.9)	2 (33.3)	14 (56.0)	
PAOD, n (%)	21 (9.3)	4 (10.3)	13 (8.4)	2 (33.3)	2 (8.0)	
CVI, n (%)	8 (3.6)	1 (2.6)	6 (3.9)	0 (0.0)	1 (4.0)	
Diabetes, n (%)	27 (12.0)	4 (10.3)	19 (12.3)	0 (0.0)	4 (16.0)	
Malignancy, n (%)	6 (2.7)	0 (0.0)	5 (3.2)	0 (0.0)	1 (4.0)	
Lung cancer, n (%)	4 (1.8)	0 (0.0)	3 (1.9)	1 (16.7)	0 (0.0)	

GOLD: Global Initiative for Chronic Obstructive Lung Disease; SD: standard deviation; FEV_1_: forced expiratory volume in 1 s; FVC: forced vital capacity; CAT: COPD Assessment Test; mMRC: modified Medical Research Council score; CHD: Coronary heart disease; PAOD: peripheral arterial occlusive disease; CVI: Cerebrovascular insult.

**Table 3:** Further COPD treatment per visit in n (%) (n = 1130 visits, 33 visits omitted due to missing GOLD group information).

	Overall	GOLD A	GOLD B	GOLD C	GOLD D	Missing (%)
	1130	196	815	10	109	
Systemic steroids	13 (1.1)	0 (0.0)	8 (1.0)	0 (0.0)	5 (4.7)	1.7
Oxygen therapy	76 (6.6)	1 (0.5)	52 (6.4)	0 (0.0)	23 (21.1)	1.6
Non-pharmacological treatment						
Sport exercise (≥twice/week)	357 (31.3)	84 (43.5)	227 (28.0)	4 (40.0)	42 (38.5)	1.8
Pulmonary rehabilitation	54 (4.7)	3 (1.5)	32 (4.0)	0 (0.0)	18 (16.5)	1.7
Vaccination (Influenza)	877 (77.3)	145 (75.1)	631 (78.4)	4 (40.0)	83 (77.6)	2.4

COPD: chronic obstructive pulmonary disease; GOLD: Global Initiative for Chronic Obstructive Lung Disease.

**Table 4:** Adherence to GOLD guidelines for treatment of stable COPD and details of non-conformity per visit (n = 1121 visits, 33 visits omitted due to missing GOLD group information, 9 visits omitted due to missing treatment information).

	Conformity, n (%)	Non-Conformity, n (%)	Missing, n (%)
Overall (n = 1121)	726 (64.8)	395 (35.2)	42 (3.6)
GOLD A (n = 195)	70 (35.9)	125 (64.1)	
no bronchodilator		20 (10.3)	
two long-acting bronchodilators		98 (50.3)	
any ICS ^a^		21 (10.8)	
GOLD B (n = 808)	541 (67.0)	267 (33.0)	
no long-acting bronchodilator		71 (8.8)	
any ICS ^a^		198 (24.5)	
GOLD C (n = 9)	7 (77.8)	2 (22.2)	
no long-acting bronchodilator		1 (11.1)	
LABA only		0 (0.0)	
LAMA plus ICS ^a^		1 (11.1)	
GOLD D (n = 109)	108 (99.1)	1 (0.9)	
no long-acting bronchodilator		1 (0.9)	
LABA only		0 (0.0)	

GOLD: Global Initiative for Chronic Obstructive Lung Disease; COPD: chronic obstructive pulmonary disease; ICS: inhaled corticosteroid; LABA: long-acting β-agonist; LAMA: long-acting muscarinic antagonist. ^a^ without documented asthma diagnosis.

## Data Availability

The data presented in this study are available on reasonable request from the corresponding author. The data are not publicly available due to restrictions in data privacy.

## Appendix A

**Table A1:** GOLD ABE-Assessment tool [29].

	Exacerbations/Year	Clinical Symptoms
Group A	≤1 moderate exacerbations (not leading to hospital admission)	mMRC < 2 CAT < 10
Group B		mMRC ≥ 2 CAT ≥ 10
Group E	≥2 moderate exacerbations or ≥1 exacerbation leading to hospital admission	independent from the clinical symptoms
